# Use of Ultrasonography for the Evaluation of Lung Lesions in Lambs with Respiratory Complex

**DOI:** 10.3390/ani15081153

**Published:** 2025-04-17

**Authors:** Alejandro Sánchez-Fernández, Juan Carlos Gardón, Carla Ibáñez, Joel Bueso-Ródenas

**Affiliations:** 1 Doctoral School, Catholic University of Valencia San Vicente Mártir, 46001 Valencia, Spain; alejandro.sanchez@ucv.es; 2 Department of Medicine and Animal Surgery, Faculty of Veterinary Medicine and Experimental Sciences, Catholic University of Valencia San Vicente Mártir, 46001 Valencia, Spain; jc.gardon@ucv.com; 3 Department of Animal Production and Public Health, Faculty of Veterinary Medicine and Experimental Sciences, Catholic University of Valencia San Vicente Mártir, 46001 Valencia, Spain; carla.ibanez@ucv.es

**Keywords:** ovine respiratory complex, ORC, thoracic ultrasound, macroscopic lung lesions

## Abstract

The ovine respiratory complex significantly affects lamb welfare and production efficiency, necessitating accurate diagnostic methods for pulmonary lesions. This study explores the relationship between clinical scoring, auscultation, ultrasonography, and macroscopic post-mortem evaluation to assess respiratory disease in 111 lambs. A standardized clinical scoring system, adapted from bovine models, evaluated ocular and nasal discharge, head tilt, cough, and rectal temperature. Auscultation categorized pulmonary sounds, while ultrasonography identified lung abnormalities, including B-lines, consolidations, pleural effusion, and abscesses. Macroscopic post-mortem examinations confirmed lesion extent. Kendall–Tau-B correlation coefficient analysis revealed significant associations between the methods (*p* < 0.01), with a high correlation between auscultation and clinical scoring *τ* of 0.634 (95% CI: 0.489 to 0.765), auscultation and ultrasonography *τ* of 0.611 (95% CI: 0.500 to 0.710), and ultrasonography and post-mortem findings *τ* 0.608 (95% CI: 0.460 to 0.731). While auscultation and clinical scoring provided useful insights, ultrasonography exhibited superior sensitivity in detecting subclinical and early-stage lesions, aligning closely with post-mortem evaluations. These findings emphasize ultrasonography as an effective tool for diagnosing respiratory disease in lambs, improving diagnostic accuracy and enabling timely interventions to mitigate disease impact and reduce antimicrobial use.

## Simple Summary

Respiratory diseases are a major health concern for lambs, affecting their well-being and growth. Veterinarians need effective methods to diagnose these diseases early to provide better care and reduce antibiotic use. This study highlights the suitability of ultrasound in diagnosing lung diseases in lambs. Traditional methods, such as lung auscultation and clinical examination, are useful but may fail to detect early or hidden infections. Ultrasound allows for earlier and more accurate detection, enabling timely treatment and reduced antibiotic use. This can improve animal welfare, minimize economic losses, and help combat antibiotic resistance. Therefore, veterinarians can adopt ultrasound as a practical and effective tool for improving respiratory disease management in lambs.

## 1. Introduction

The specific management practices and zootechnical factors implemented on a lamb fattening farm play a crucial role in animal health outcomes, particularly in the epidemiology of the ovine respiratory complex (ORC) [1]. In this sense, ORC is among the leading causes of mortality, welfare decline, and economic losses in farms and feedlots [2,3,4]. ORC is a multifactorial disease influenced by host-related factors, environmental conditions, management practices, and etiological agents, with the combination of these elements being recognized as predisposing factors [4,5]. Consequently, a comprehensive understanding of ORC dynamics and the accurate identification of common disease manifestation patterns are essential for a thorough examination of this pathology. Additionally, animal age and the location of the fattening process are critical factors directly influencing ORC epidemiology. In this regard, disease management protocols and scoring systems have proven to be effective and cost-efficient tools [6] for identifying and treating clinically affected animals [7,8]. These strategies would promote respectful, mindful, and reduced use of antibiotics on farms [9,10], thereby mitigating the risks associated with the development of antibiotic resistance. Scoring systems provide a standardized methodology for the physical examination of farm animals. However, the accuracy of certain clinical assessment criteria may vary [11,12]. ORC presents nonspecific clinical symptoms, including fever, anorexia, dyspnea, coughing, and nasal and ocular discharge, which may manifest partially or completely [5]. Three clinical forms of ORC can be distinguished based on their presentation: hyperacute, acute, and chronic [5,13]. *Pasteurella multocida*, *Mycoplasma* spp. *Mannheimia haemolytica*, and *Bibersteinia trehalosi* are the primary microorganisms isolated in ORC [5,14,15], but *Escherichia coli*, *Trueperella pyogenes*, *Staphylococcus* spp. *Streptococcus* spp., and *Pseudomonas* spp. are also linked to respiratory disease in small ruminants [5,16]. The clinical evaluation of symptoms in field conditions can be complemented with auscultation, which allows the detection of variations in respiratory rate and respiratory sounds [8,17]. However, no scientific evidence has established a direct relationship between the etiology of respiratory disease and auscultatory alterations [18]. The subjective nature of these assessments and the absence of a reference method for detecting respiratory disease in live animals limit the diagnostic value of this approach, highlighting the need for further refinement. Post-mortem examinations are techniques that enable the visualization, localization, and description of lesions in affected organs, and bronchopneumonia is the primary lesion observed in lambs affected by ORC [19].

Imaging diagnostic techniques, especially ultrasonography, are increasingly finding applications in farm animals [20,21,22]. Pulmonary ultrasonography is a widely used technique in dairy calves [23,24,25], proving useful for diagnosing lung lesions and predicting the prognosis of calves with bovine respiratory disease (BRD) [25,26]. Ultrasonography has been described as a reliable technique for assessing the nature and extent of superficial lung pathology in sheep [27,28]. This imaging modality allows for the visualization of abnormal lung tissue resulting from inflammatory processes, thus providing insight into the severity and extent of lung damage. In ultrasonographic images of healthy lung tissue, artifact lines appear as parallel lines along the lung surface due to total air reflection (A-lines) [8,25,29,30]. Studies show that ultrasonography can detect a wide range of pulmonary and pleural conditions in both young and adult animals, including pulmonary edema, consolidation, abscesses, necrosis, pleural effusion, pneumothorax, and atelectasis [31,32,33]. Furthermore, ultrasonography serves as a valuable tool for identifying at-risk populations, monitoring the prevalence and severity of respiratory disease, and assessing the impact of management changes—such as ventilation modifications, vaccinations, or weaning—on animal health [27]. Additionally, it has been used for daily monitoring of an animal’s response to treatment, thereby aiding in the evaluation of therapeutic efficacy [34,35,36]. The standard on-farm ultrasonographic examination protocol is both rapid and straightforward for trained observers [37], making ultrasonography not only a precise diagnostic tool but also a practical one [8]. Thus, the hypothesis driving this study is that thoracic ultrasonography is a valid diagnostic tool for detecting pulmonary disease in lambs. The primary objective of this study was to evaluate the relationship between different diagnostic tools such as auscultation, clinical scores, pathological anatomy, and ultrasonography in assessing lung disease in lambs, with the aim of determining the suitability of ultrasonography as a complementary diagnostic tool in field conditions.

## 2. Materials and Methods

### 2.1. Animal Facilities and Management

The study was conducted on a Lacaune breed dairy sheep farm located in Catadau, Valencian Community, Spain. A total of 111 Lacaune lambs (male and female) were included in the study. Following parturition, the lambs were separated from their dams and transferred to artificial rearing facilities. During this period, they were fed a diet consisting of a commercial milk replacer (ELVOR 63: crude protein, 24%; fat, 24%; fiber, 5%; ash, 7%; calcium, 0.9%; sodium, 0.45%; phosphorus, 0.75%), cereal straw, and commercial starter feed (Lactoiniciacor Nanta: crude protein, 18%; crude fiber, 4%; fat, 3%; ash, 6.9%; starch and sugars, 43%). Milk replacer consumption gradually declined until the lambs reached 40 days of age, at which point they were weaned upon reaching an approximate body weight of 17 kg. After weaning, the lambs were transferred to fattening facilities, where they were raised until 70 days of age, reaching an approximate weight of 28 kg. During the fattening phase, the animals were housed in pens measuring 3 × 13 m, with approximately 40 lambs per pen. Throughout this period, they were provided ad libitum access to cereal straw and a concentrated fattening feed (Nantacor Intensive Fattening Nanta: crude protein, 17.5%; fiber, 4.3%; fat, 3%; ash, 42%; starch and sugars, 42%). Animal selection and tests were developed 12h before transportation to the slaughterhouse. The animals selected for the study were those that exhibited pathologies that could lead to growth delays or locomotor, enteric, or respiratory issues, yet were still fit for transportation to the slaughterhouse, where samples were collected for pathological anatomy analysis (see Section 2.2).

### 2.2. Diagnostic Methods Employed, Corresponding Animal Procedures and Scores

The clinical score (SClinic) diagnostic method used for lambs was the Wisconsin Calf Respiratory Score (WCRS) [38]. A total of six clinical signs were evaluated: ocular discharge (OD): serous, mucous, or purulent discharge, unilateral or bilateral, nasal discharge (ND): Serous, mucous, or purulent nasal discharge, unilateral or bilateral, head tilting (HT), COUGH: impulsive and audible air exhalation and rectal temperature (RT): above or below 39.5 °C. Each clinical sign was scored on a scale from 0 to 3, where 0 indicated the absence of symptoms or normal temperature values, and 3 represented the most severe alteration (Table 1).

The final SClinic for each animal was calculated as the sum of the points assigned to each clinical sign and severity level. The final score ranged from 0 to 15, where 0 indicated no symptoms or normal temperature, and 15 represented the highest level of alteration. The scores were subsequently grouped into the following categories: 0–2 (score 0), 3–7 (score 1), 8–11 (score 2), and 12–15 (score 3, Table 2). This categorization is used to group animals based on their clinical signs and severity, allowing for a systematic classification of the level of illness.

Auscultation (SAusc) was performed on lambs within the farm facilities using a stethoscope (3M Littmann Classic III, 3M Medica, St. Paul, MN, USA). Both pulmonary hemithoraxes were systematically auscultated, ensuring evaluation of all pulmonary lobes. The assessment started with an evaluation of the left hemithorax, adhering to a systematic protocol. The evaluation covered the cranial and caudal lobes: specifically, the cranial area of the cranial lobe between the 2nd and 3rd intercostal spaces and the caudal area above the sternum between the 4th and 5th intercostal spaces. The caudal lobe was also assessed in the ventral area between the 7th and 9th intercostal spaces and the dorsal area between the 8th and 10th intercostal spaces. After the left hemithorax, the right hemithorax was assessed using an identical protocol. For the cranial lobe, two specific regions were assessed: the cranial area, examined through the 2nd to 3rd intercostal spaces, and the caudal area, situated above the sternum between the 4th and 5th intercostal spaces. The middle lobe was evaluated in the region between the 5th and 6th intercostal spaces, aligned dorsally with the thoracic midline. The caudal lobe was similarly scrutinized in two distinct zones: the ventral area, spanning the 7th to 9th intercostal spaces, and the dorsal area, between the 8th and 10th intercostal spaces, using the diaphragm as a reference point. The classification of lambs based on auscultatory findings was systematically recorded as follows: normal lung sounds were denoted as 0, bronchial breath sounds as 1, the presence of crepitations, wheezes, or rales as 2, and the absence of lung sounds as 3 (Table 2). Once the auscultation was completed, the final score was assigned based on the most severe finding diagnosed.

**Figure 1:**
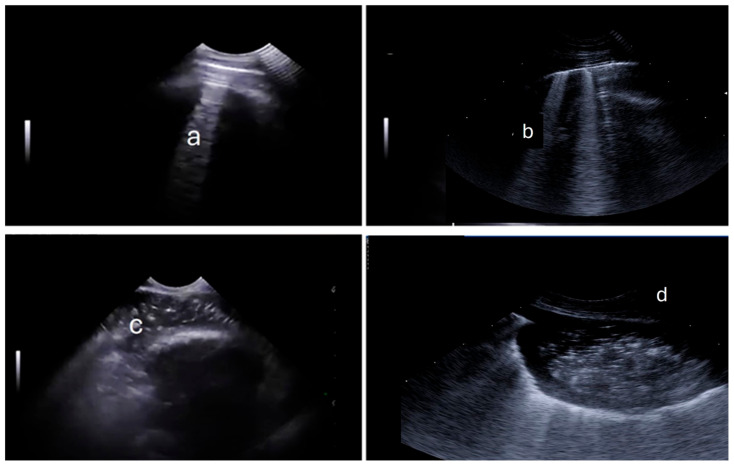
Pulmonary ultrasound of lambs; (**a**): indicating A-lines; (**b**): B-lines; (**c**): consolidation; (**d**): abscess.

Ultrasound examination was performed using an Esaote MyLab One Vet ultrasound system (Esaote España, Barcelona, Spain) equipped with a micro convex probe (SC3123). The reference frequency was set at 10 MHz, with an imaging depth of 8 cm. A hydroalcoholic gel was applied to enhance ultrasound transmission and optimize image visualization. The examination was conducted in a dimly lit room, with the lamb gently placed in lateral recumbency on a padded surface. The ultrasound evaluation began with the left hemithorax, followed by the right. The cranial, middle, and caudal lobes were examined sequentially. The cranial lobe was visualized using the ultrasound probe placed in the 2nd to 3rd intercostal spaces, allowing for the visualization of pulmonary arteries and veins as anatomical landmarks. For the right middle lobe, the ultrasound examination involved two areas. The ventral area was assessed by positioning the probe above the sternum within the 4th or 5th intercostal space, using the heart as a reference. The dorsal zone was examined between the 5th and 6th intercostal spaces. The caudal lobe examination included two points. In the ventral area, the probe covered the 7th to 9th intercostal spaces, with the liver used as a reference. The dorsal area was examined between the 8th and 10th intercostal spaces, using the diaphragm as a landmark. Findings of the ultrasounds (Figure 1) were documented and categorized into the following classifications: A-lines, B-lines, consolidation (CON), pleural effusion (PE), and abscess (ABS). After the examination, results of the complete exam were summed and classified into the ultrasound score (Sult) as follows: Score 0: lambs with normal lungs. Score 1: >5 sites displaying B-lines with no CON. Score 2: >5 sites displaying B-lines and <5 sites with CON. Score 3: >5 sites displaying CON, presence of PF, or ABS (Table 2).

**Figure 2:**
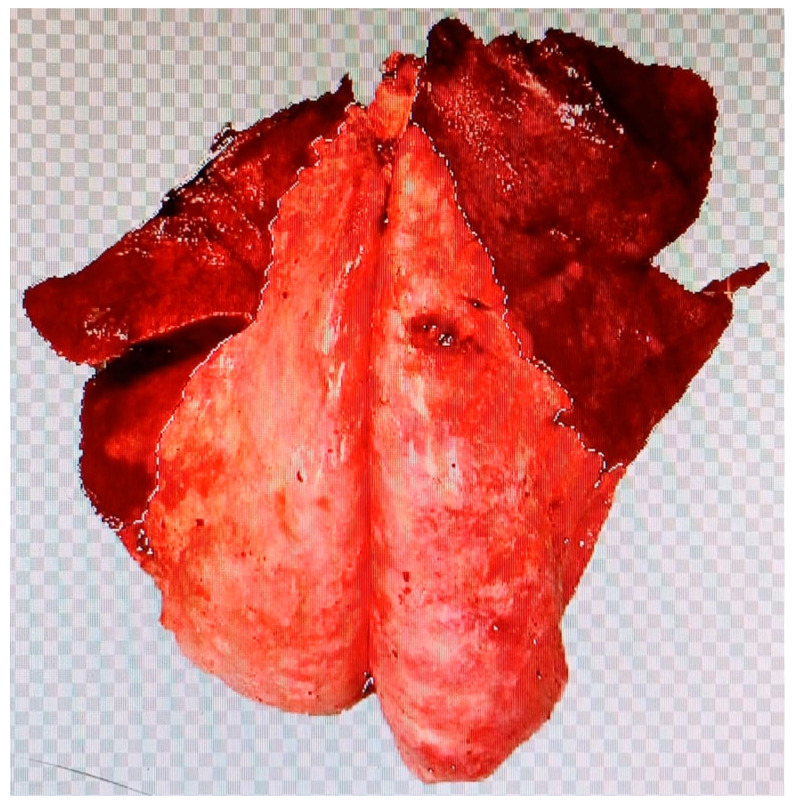
Lung lesion area marked for quantification (percentage of affected area).

Post-mortem macroscopic inspection was conducted following slaughter at the abattoir twelve hours after being assessed for ORC using SAusc, SClinic, and SUlt. Pulmonary lesions were photographed and described according to their distribution, margins, shape, color, size, texture, consistency, and extent. Using Adobe^®^ Photoshop^®^ CC2023 (24.0), the affected lung areas were marked and the lesion area was quantified (Figure 2). Pneumonias were classified based on the extent of lung consolidation, following a 0–3 scoring system, referred to as the post-mortem score (SPost, Table 2).

### 2.3. Statistical Analysis

To assess the relationship between different diagnostic methods and their scoring distributions among lambs, statistical analyses were conducted using IBM SPSS Statistics software (V29.0.2.0). Frequency tables were used to examine the distribution of scores across diagnostic methods.

The Kendall–Tau-B correlation coefficient was employed to measure the association between different diagnostic methods given the ordinal nature of the data. The strength of the correlations was categorized as follows: low (0.1–0.3), moderate (0.3–0.5), high (0.5–0.7), and very high (>0.7). Statistical significance was set at *p* < 0.01.

## 3. Results

In this study, a total of 111 lambs were assessed using various diagnostic methods, revealing varied scoring distributions. For SAusc, 30.63% of the lambs (34/111) were scored as 0, another 30.63% (34/111) as score 1, the same proportion 30.63% (34/111) as score 2, and a smaller fraction, 8.11% (9/111), as score 3. In the SClinic evaluations, 32.43% of the lambs (36/111) received a score of 0, 34.23% (38/111) a score of 1, 23.42% (26/111) a score of 2, and 9.90% (11/111) a score of 3. SUlt assessments showed that 24.32% of lambs (27/111) were scored as 0, 19.82% (22/111) as 1, 23.42% (26/111) as 2, and the highest severity score, 3, was given to 32.43% (36/111) of the lambs. For SPost, 34.23% (38/111) were scored as 0, 19.82% (22/111) as 1, 18.02% (20/111) as 2, and 27.93% (31/111) as 3 (Table 3).

Subsequent analysis using Kendall’s *τ* correlation coefficient quantified the relationships between these diagnostic methods, all of which were statistically significant with *p*-values below 0.01. The correlation between SAusc and SClinic was notably strong, with a *τ* of 0.634 (95% CI: 0.489 to 0.765). SAusc and SUlt also showed a significant correlation with a *τ* of 0.611 (95% CI: 0.500 to 0.710). The correlation between SAusc and SPost was moderate, evidenced by a *τ* of 0.407 (95% CI: 0.246 to 0.546). Additionally, SClinic and SPost also demonstrated a moderate correlation *τ* of 0.480 (95% CI: 0.331 to 0.617). Strong correlations were observed between SClinic and SUlt, and SUlt and SPost, with *τ* values of 0.611 (95% CI: 0.497 to 0.722) and 0.608 (95% CI: 0.460 to 0.731), respectively (Table 4).

## 4. Discussion

Sheep are highly susceptible to respiratory diseases [39] such as ORC, which is one of the leading causes of mortality in fattening lambs [5]. As with other diseases, an early and accurate detection method is crucial for ensuring animal welfare [20], reducing the reliance on antibiotics or other medications [8]. Not only does this approach enhance animal welfare, but health management also improves farm profitability [40] and ensures the safety of the products derived. Antibiotic residues in animal-derived food can lead to direct toxicity [41]; moreover, even low levels of antibiotic exposure may disrupt microflora, contribute to the development of antimicrobial-resistant strains [42], and result in therapeutic failures [43]. In feedlot calves, pulmonary ultrasonography is increasingly regarded as a simple and effective technique for diagnosing respiratory diseases. It facilitates field-based assessments and provides critical information for making informed decisions regarding the appropriateness of antibiotic treatments and other management strategies [8]. The results of this study confirm that, similarly to observations in calves, pulmonary ultrasonography can be a convenient technique for diagnosing ORC in fattening lambs.

Traditionally, the diagnosis of ORC in the field has relied on clinical symptom assessment, occasionally supplemented with auscultation. However, a definitive diagnosis of the disease usually is based on a post-mortem examination of deceased or euthanized animals [8,44]. For this study, the auscultation score (SAusc) was adapted based on the most frequently detected pulmonary sounds in the field [17,18,45]. On the one hand, the distribution of animals across SAusc scores 0, 1, and 2 was similar. On the other hand, low prevalence (8.11%; 9/111) was observed for SAusc score 3, whereas higher prevalences were found for score 3 in SUlt (32.43%; 36/111) and SPost (27.93%; 31/111). Additionally, the correlation between SAusc and SPost *τ* of 0.407 (95% CI: 0.246 to 0.546) suggests that auscultation alone is insufficient for diagnosing pulmonary lesion. These results could indicate difficulty in differentiating disease severity; in this study, the frequency distribution for scores 0, 1, and 2 was the same, indicating that animals without normal sounds, those with bronchial sounds, and those with crepitations, wheezing, and rales were diagnosed in the same proportion. In this sense, some authors have pointed out that the auscultation technique suffers from a lack of specificity due to the difficulty encountered in correlating pulmonary sounds that are considered pathological with specific lesions found in pathological anatomy [17,18]. Other authors suggest that the sensitivity and specificity of pulmonary sound interpretation can be affected by the operator’s expertise, environmental factors, skin and wool interference, and the misinterpretation of miscellaneous sounds, such as gastrointestinal borborygmi or ruminal noises [17,18,45]. Moreover, auscultation has been noted for its limitations in detecting and localizing abscesses [17,18]. In any case, auscultation is a cost-effective and user-friendly technique that, when employed under calm conditions by experienced technicians, can serve as an excellent tool for initial screening or evaluation of animals with moderate or no symptoms. This makes it particularly useful during the early stages of the disease.

In this study, the SClinic used was the Wisconsin Calf Respiratory Score (WCRS) [38], which considers species-specific factors, size, and production system characteristics. SClinic included clinical signs described in cattle OD, ND, HT, cough and RT, all of which were also observed in the lambs of this study and were associated with ORC. The bovine respiratory scoring system is a widely used diagnostic method on farms, providing objective information on disease progression [6,8,11,12,29,38,46]. A significant correlation was found between SAusc and SClinic *τ* of 0.634 (95% CI: 0.489 to 0.765), with similar prevalence distributions in disease detection and classification across scores, suggesting that pulmonary sounds and clinical symptoms are clear indicators of respiratory disease in affected animals. Moreover, the correlation between SClinic and SPost *τ* of 0.480 (95% CI: 0.331 to 0.617) supports the reliability of clinical scoring in disease diagnosis. Considering macroscopical pathological anatomy as a reference to describe lesions [8,44], these findings underscore the significant utility of employing this type of clinical scoring. In addition to its practicality and ease of use by technicians, it allows for the assessment of the severity of the disease state in individuals across a farm. It is for this reason that such clinical scoring systems have been widely adopted in calves. The results of our study confirm that these scores would be equally valuable on fattening lamb farms.

SClinic showed a high correlation with SUlt *τ* values of 0.611 (95% CI: 0.497 to 0.722). Similar correlation values (rsp = 0.70; *p* < 0.01) have been observed in cattle between SClinic and SUlt [8]. Regarding score distribution, 32.43% (36/111) of the lambs were categorized as score 0 (asymptomatic) according to SClinic, whilst fewer only 27/111 (24.3%) scored 0 according to Sult. A similar result was observed by Scott et al. [18]. This last result suggests that ultrasonography enables early diagnosis of subclinical cases, detecting pulmonary alterations in initial stages even in the absence of clinical signs [29,47]. Early diagnosis of BRD is crucial for improving treatment success rates and minimizing the risk of treatment failure [48]. Also, ultrasonography would allow for a more comprehensive examination by locating, quantifying, and classifying lesions, which would provide more information for decision-making. In this sense, Hussein et al. [29] reported that combining clinical and ultrasonographic diagnosis significantly improves diagnostic accuracy in calves and buffaloes. Additionally, in our study, ultrasonography identified animals with more severe pulmonary conditions.

In this study, a complete individual macroscopic post-mortem examination was performed, describing the pulmonary macroscopic lesions according to their distribution, margins, shape, color, size, texture, texture, consistency, and extent. The most frequent finding was the presence of reddish-brown discoloration in the cranioventral lobes, consistent with bronchopneumonia caused by ORC [19,44] The results of our study revealed a high correlation between SUlt and SPost *τ* values 0.608 (95% CI: 0.460 to 0.731), reinforcing the superior capability of ultrasonography in differentiating and identifying pulmonary lesions compared to auscultation and clinical diagnosis. Also, other studies in calves have reported high correlations between ultrasonographic findings and pathological anatomy [8,49,50]. The results of the present study show that SUlt and SPost were statistically equivalent in terms of how they classify the severity of conditions in lambs. So, it can be concluded that ultrasonography and macroscopical pathological anatomy are nearly equivalent in their capabilities to diagnose ORC. Moreover, the efficacy of ultrasonography has been extensively documented in diagnosing a variety of pulmonary conditions in adult small ruminants, such as mycoplasmosis, viral diseases such as OPA and Lentivirus disease, parasites, and pseudotuberculosis [14,18,27,32,47,51,52,53]. Despite its extensive applicability, ultrasonography does exhibit certain limitations, as it may not effectively detect deep-seated lesions if the pleural region and the superficial lung parenchyma are well-aerated. It would also be less effective in detecting lesions in mediastinal nodules, lung lesions adjacent to the spine, or those within the accessory pulmonary lobe. However, these limitations are generally not a concern in fattening lambs, where ultrasonography would be a good tool in the early, regular, and accurate diagnosis of respiratory diseases. Although further studies are required to confirm this hypothesis, pulmonary ultrasonography provides valuable insights into the localization, severity, and extent of lesions and, combined with an understanding of the disease epidemiology, this information would assist in etiological diagnosis and thus in the establishment of necessary strategies for disease control.

## 5. Conclusions

Lung ultrasonography provides a more accurate evaluation of the severity, extent, and localization of lesions compared to traditional auscultation and clinical examination, and it also demonstrates a significant positive correlation with post-mortem evaluations. Furthermore, the ability of ultrasonography to perform these assessments on live animals could represent an advantage over pathological anatomy. Pulmonary ultrasonography thus enhances our ability to diagnose and manage respiratory diseases effectively in a timely and non-invasive manner. The benefits of ultrasonography, when combined with other diagnostic tools and a thorough understanding of the disease’s epidemiology, lead to more comprehensive diagnoses, facilitating immediate decision-making and implementation in a farm setting. Further studies are necessary to establish a relationship between lesions visualized by ultrasonography and the etiology responsible of these lesions.

## Figures and Tables

**Table 1:** Summation of Clinical Symptom Evaluation for the Diagnosis of ORC (Clinical Score).

Diagnosis	Score			
	0	1	2	3
OD	No	Mild unilateral serous discharge	Bilateral serous discharge	Bilateral mucosal discharge
ND	No	Mild unilateral serous discharge	Unilateral serous discharge	Bilateral mucosal discharge
HT	No	Head movement	Unilateral head drooping	Bilateral head drooping
COUGH	No	Induced and single cough	Induced and multiple or Spontaneous and occasional	Spontaneous and multiple
RT	<39.49 °C	39.5–39.89 °C	39.9–40.49 °C	≥40.5 °C

OD: ocular discharge, ND: nasal discharge, HT: head tilting and RT: rectal temperature.

**Table 2:** Scoring system used in lambs for each of the diagnostic methods performed: clinical (SClinic), auscultatory (SAusc), ultrasonographic (SUlt^1^), and post-mortem macroscopic lung evaluations (SPost).

	SAusc	SClinic	SUlt^1^	SPost
0	Normal lung	Sumatory between 0–2	Normal lung	<10%
1	Bronquial breath sounds	Sumatory between 3–7	>5 B-lines, no CON	10–20%
2	Crepitations, wheezing and rales	Sumatory between 8–11	>5 B-lines, <5 CON	20–30%
3	Absence of lungs sounds	Sumatory between 12–15	>5 CON, PF o ABS	>30%

B-lines: comet tails, CON: consolidation, PF: pleural effusion and ABS: abscess.

**Table 3:** Frequency distribution of scores for each diagnostic method.

Evaluation	Score 0	Score 1	Score 2	Score 3	Total
SAusc	34	34	34	9	111
SClinic	36	38	26	11	111
SUlt	27	22	26	36	111
SPost	38	22	20	31	111

SAusc: auscultation score; SClinic: clinical score; SUlt: ultrasonography score; SPost: pathological anatomy score.

**Table 4:** Correlation matrix of clinical score (Sclinic), auscultatory (SAusc), ultrasonographic (SUlt1), and post-mortem macroscopic lung evaluation (SPost). The upper triangle in grey corresponds to 95% confidence interval. The lower triangle corresponds to the values of the correlations.

	SAusc	SClinic	SUlt	SPost
SAusc	1.00	0.500–0.710	0.500–0.710	0.246–0.546
SClinic	0.634	1.00	0.497–0.722	0.331–0.617
SUlt	0.611	0.611	1.00	0.497–0.722
SPost	0.407	0.480	0.608	1.00

SAusc: auscultation score; SClinic: clinical score; SUlt: ultrasonography score; SPost: pathological anatomy score.

## Data Availability

The data that supports the findings of this study are available from the corresponding author upon request.
